# Diurnal differences in urine flow in healthy young men in a light-controlled environment: a randomized crossover design

**DOI:** 10.1186/s40101-023-00346-z

**Published:** 2023-11-17

**Authors:** Hiromitsu Negoro, Isuzu Nakamoto, Sayaka Uiji, Yoshiko Matsushima, Bryan J. Mathis, Dominika Kanikowska, Tomoko Wakamura

**Affiliations:** 1 Department of Urology, Institute of Medicine, University of Tsukuba, Ibaraki, 305-8575 Japan; 2 Department of Gerontological and Home Healthcare Nursing, Graduate School of Medicine, Tohoku University, Miyagi, Japan; 3 Human Health Sciences, Graduate School of Medicine, Kyoto University, Kyoto, Japan; 4 International Medical Center, University of Tsukuba Affiliated Hospital, Ibaraki, Japan; 5 Department of Pathophysiology, Poznan University of Medical Sciences, Poznan, Poland

**Keywords:** Bladder, Light, Maximum urine flow, Qmax

## Abstract

**Background:**

Older men often experience nocturnal urination difficulties, reflected by diurnal differences in maximum urine flow (Qmax). Since lower urinary tract symptoms and pathological comorbidities are frequent in older men, it remains unclear whether this diurnal variation is a physiological or pathological phenomenon. Our aim was to quantify the diurnal variability of Qmax in healthy young participants under varying daylight conditions in a stable environment to discern potential underlying causes of nocturnal urination difficulties.

**Methods:**

Twenty-one healthy young men were recruited in a 4-day study utilizing daytime (08:00–18:00) exposure with two light conditions in randomized order: dim (< 50 lx) or bright (~2500 lx). Day 1 was for acclimation, and urine flow was assessed from day 2. The participants urinated *ad libitum* during day 2 and then at fixed 3–4-h intervals thereafter (days 3–4). Regular urination Qmax at late night (04:00) on day 4 was compared with the nearest voided volume during daytime of day 3 (mDay).

**Results:**

Morning Qmax scores (after bed—11:00) on day 2 were significantly lower than evening (17:00—before pre-sleep) in bright conditions and those of daytime (11:00–17:00), evening (17:00—before pre-sleep), and pre-sleep in dim conditions. Pre-sleep Qmax during the *ad libitum* period was significantly higher in dim than bright conditions. Late-night Qmax values (04:00) on day 4 were significantly lower than Qmax scores of mDay on day 3 in both light conditions.

**Conclusions:**

Healthy young men had a clear diurnal Qmax difference that decreased during late night and morning. In addition, the pre-sleep Qmax values in dim daylight were significantly higher than in bright daylight. Taken together, we conclude that late-night and morning decreases in Qmax are an instinctive physiological phenomenon in humans, and the diurnal difference of Qmax can be influenced by daylight conditions.

**Supplementary Information:**

The online version contains supplementary material available at 10.1186/s40101-023-00346-z.

## Background

Men over 50 may experience difficult nocturnal or morning urination, and objective assessment of urinary function in this population includes evaluation of maximum urine flow rate (Qmax) by uroflowmetry. Qmax, which represents the peak flow rate during urination, is a pivotal metric in urology [1]. Expressed in milliliters per second (ml/s), it offers insights into how efficiently the lower urinary tract (including bladder and urethra) functions. A Qmax of less than 15 ml/s is generally considered abnormal [1, 2]. Diurnal differences in Qmax have been reported in both older men with lower urinary tract symptoms and hospitalized patients in the urology department [3–8]. However, older men with lower urinary tract symptoms often have pathological comorbidities (e.g., benign prostatic obstruction, overactive bladder, or underactive bladder) that complicate determination of diurnal Qmax differences due to other age- or disease-specific conditions. Consequently, erroneous assumptions that difficulties with nocturnal or morning urination are indicative of deteriorating pathological conditions could persist. However, if Qmax variation is proven to be a physiological phenomenon, patients, medical staff, and the general public could recognize such diurnal differences as natural, and this would significantly reduce anxiety associated with potential pathological issues.

What might be the possible root of this diurnal difference in Qmax? Physiological functions, such as urine production, body temperature, blood pressure, and sleep/awake cycles, are controlled via diurnal rhythms mediated by the endogenous circadian clock [9]. Melatonin, a chronobiotic hormone released from the pineal gland under the regulation of light exposure duration and timing, plays a key role in modulating the cyclic function of this system [10]. Since the circadian clock system is strongly believed to control urination rhythm and functional bladder capacity [11, 12], any observed diurnal differences in Qmax are presumed to be similarly cyclical. In spite of this, the effect of circadian rhythms on Qmax variations has yet to be explored in young healthy men without urinary comorbidities in a controlled environment. It is crucial to conduct experiments in a stable, controlled environment when investigating physiological phenomena, especially those as sensitive as urination, which can be influenced by both environmental and emotional factors. Light stimuli, acting as cues for the central master clock, have a significant impact on diurnal physiological variations. In light of this, we chose to examine Qmax under two distinct but physiologically relevant conditions: one mirroring the bright illumination typical of our daily rhythms and another that minimizes the pronounced external influence of light [13].

Our primary objective was to investigate the underlying cause of urinary difficulties at night or early in the morning often observed in elderly men. To achieve this, we examined potential diurnal Qmax variations in healthy young men, with a focus on the effects of inherent diurnal regulation.

## Participants and methods

**Fig. 1:**
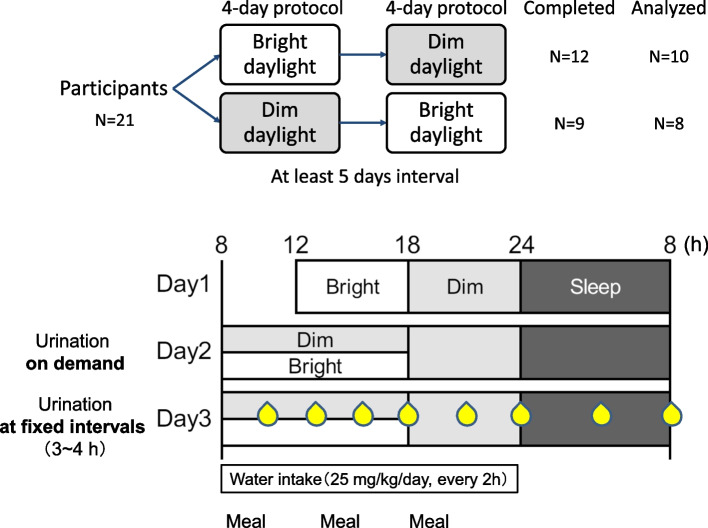
A schema of the study protocol

This study was performed concurrently with the other experiments previously described [13]. All participants provided their informed written consent to take part in this study. The study was endorsed by the Ethics Committee of Kyoto University (no. C1449) in accordance with the principles of the Declaration of Helsinki and registered in the database of the University Hospital Medical Information Network (UMIN000038350). From November 2019 to February 2020, 21 healthy young men (21–27 years old) were recruited from Kyoto University. Starting 5 days prior to the examination, participants were instructed to go to bed at 24:00 (midnight) and wake up at 8:00 while abstaining from alcohol, ensuring a regulated lifestyle. Sleep habits prior to the experiment were confirmed by sleep diaries written by the participants. They participated in a 4-day study (4-day study ×2) with random order of exposure to two light conditions during the day (08:00–18:00), dim and bright (Figure 1). In the experiment, we set the dim daylight condition to less than 50 lx (10 μW/cm^2^, 2822 K, 14 lx melanopic equivalent daylight illuminance [EDI]). This was based on our previous study, which demonstrated a delay in melatonin secretion under this condition [14]. On the other hand, the bright daylight condition was set to approximately 2500 lx (734 μW/cm^2^, 3923 K, 1442 lx melanopic EDI) because, in earlier findings, it was not 1500 lx but 2500 lx that influenced nocturnal melatonin secretion [15, 16]. Light intensity was gauged vertically at eye level by a researcher in a seated position, using a Lighting Passport spectrometer from Asensetek Inc., Taipei, Taiwan. The light sources utilized were fluorescent lamps, which illuminated the experimental room through a pseudo-window [13]. Melanopic EDI was calculated using a new SI-compliant measurement system (CIE S 026:2018) [17]. The study room had a controlled air temperature (25 ± 2 °C) and humidity (55 ± 5%). Rectal temperature was continuously recorded during the experiment. All urination was counted, and Qmax was evaluated by P-Flowdiary® (Micronix Inc., Kyoto, Japan). On the second day of the experiment (day 2), participants were allowed to urinate *ad libitum* but directed to urinate regularly at fixed intervals (every 3–4 h) thereafter (day 3).

In the free urination protocol, the specific act of urination was separately categorized as pre-sleep, considering its unique occurrence at the end and later part of the day. The time intervals of 11:00–17:00 and 17:00—before pre-sleep were adopted to represent approximately 6-h intervals, equating to a quarter of a 24-h day, capturing the daytime and evening periods, respectively. As no participants urinated during nighttime, the period was designated as “after bed~11.” Consequently, for analyzing morning urination, the timeframe was set until 11:00. When there were multiple urination events, the average value was calculated. Regarding the urine production rate, the volume from a single urination was divided by the time elapsed between urinations to determine the urine production rate per time unit [18]. This rate was further broken down into intervals: from wake-up to 11:00, 11:00 to 17:00, 17:00 to before sleep, and the sleep period, given that urine production continues during nighttime sleep. For urination frequency, we employed a similar approach as that used for the urine production rate. Qmax is known to correlate with the amount of voided volume [19], and there are significant individual differences in this respect [20].

In addition to the free urination protocol, we utilized and adopted a regular urination protocol experiment by Nakamoto et al. [13] However, the Nakamoto study was primarily focused on changes in diurnal urine volume and urine electrolytes, while the present study was not intended to elucidate complete and comprehensive diurnal variations of Qmax. Instead, our objective was to compare Qmax data from 4 a.m. urination with daytime data, aiming to examine periods not observed during spontaneous urination. It is crucial to note that regular urination is not prompted by a natural urge, and the bladder is not fully filled during daytime urination. Additionally, while Qmax is known to correlate with the amount of voided volume [19], there are significant individual differences in this respect [20]. Therefore, to ensure an accurate comparison of Qmax values, we designed a protocol to compare these data with daytime urination of a similar volume from a clinical standpoint of nocturnal urination difficulties. Considering these variations, the Qmax at 04:00 on the fourth day was compared with the closest amount of voided volume during the day from 11:00 to 18:00 (matched day: mDay).

Sleep time was fixed from 24:00 to 08:00. Identical meals (700 kcal each: 60% carbohydrates, 25% fat, and 15% protein; 3.8 g salt) were provided to participants at 08:00, 13:00, and 18:00. Participants consumed a total of 25 ml/kg body weight of water per day in doses given every 2 h between 08:00 and 24:00. Not required to follow a strict daily routine, but to avoid extreme measurements, participants were discouraged from strenuous exercise or napping. The exclusion criteria for this study were the presence of sleep disturbances as measured by the Pittsburgh Sleep Quality Index (PSQI) [21] and extreme morning or evening sleep events as measured by the Morningness-Eveningness Questionnaire [22]. According to Cornell Medical Index values [23] and International Prostate Symptom Scores [24], all participants were in good health and did not have urinary problems. None of them smoked cigarettes, took medication, had food allergies, or travelled across time zones in the 3 months leading up to the start of the experiment.

### Statistical analyses

For the comparison between time periods, we used one-way ANOVA with Dunnet’s *post hoc* test. Two-way repeated measures ANOVA was used to compare differences between the dim and bright daylight conditions, while timepoints were compared with Sidak’s *post hoc* test. Symbols or bars represent mean ± standard error of the mean. All statistical analyses were performed using the commercially available software package GraphPad Prism v8.43 (GraphPad Software Inc., CA, USA).

## Results

**Fig. 2:**
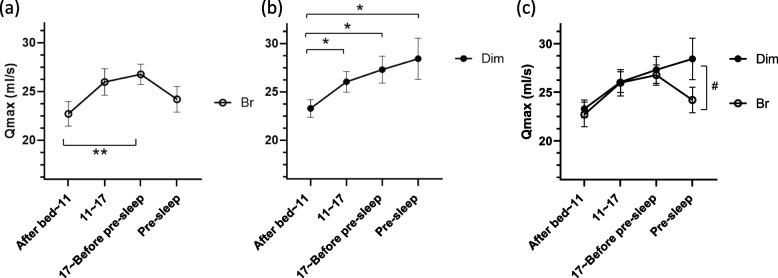
Diurnal differences in maximum flow rates (Qmax) during the free urination period in bright daylight conditions (white circle) in **a**, in dim daylight conditions (filled circle) in **b**, and the merged graph in **c** (*p < 0.05, **p < 0.01 by Dunnett’s multiple comparison test, one-way ANOVA; #*p* < 0.05 by two-way repeated measures ANOVA followed by Sidak’s multiple comparison test). Error bars represent SEM. Br indicates bright daylight conditions

**Fig. 3:**
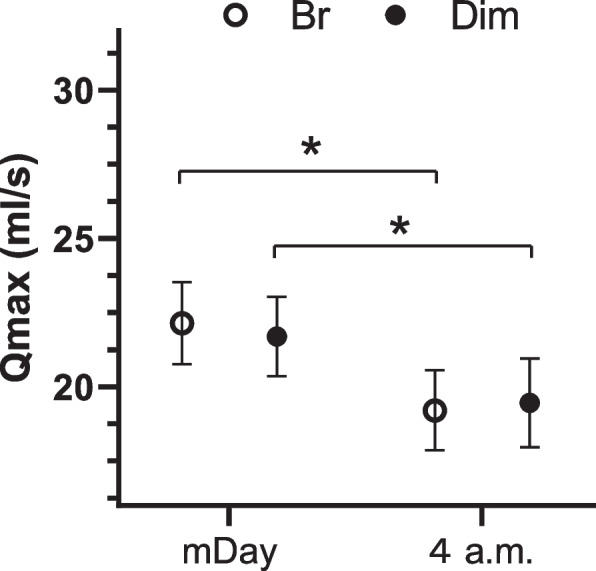
Diurnal differences in maximum flow rates (Qmax) during the regular urination period in bright and dim daylight conditions (*p < 0.05 by two-way repeated measures ANOVA followed by Sidak’s multiple comparison test). mDay, matched day, indicating closest amount of volume voided per micturition in the daytime compared with 4 a.m. Error bars represent SEM. Br indicates bright daylight conditions

The mean ± standard deviation age, BMI, PSQI, and MEQ of the participants were 23.9 ± 1.6 years old, 21.6 ± 1.5 kg/m^2^, 4.2 ± 1.7, and 49.2 ± 8.0, respectively. The participants were generally of average build and height and believed to have regular lifestyles without sleep disorders, making them suitable for this experiment. Three participants were excluded due to lack of Qmax data. In the remaining participants, morning Qmax (after bed—11:00) values were significantly lower than evening Qmax (17:00–23:00) values obtained in bright daylight under free urination conditions (*p* < 0.01) (Fig. 2a) and at 11:00–17:00, 17:00–23:00, and pre-sleep timepoints in the dim daylight experiment (*p* < 0.05 each) (Fig. 2b). The pre-sleep Qmax values in dim daylight were significantly higher than in bright daylight (*p* < 0.05) (Fig. 2c). Urinary frequencies and urine production rates during the sleep period were significantly lower than in the morning (wake-up—11:00), daytime (11:00–17:00), and evening (17:00—before sleep) periods in both bright and dim daylight (Supplementary Fig. 1). The volumes voided per micturition or average flow rates during sleep period were not significantly different in both bright and dim daylight (Supplementary Fig. 2). An analysis was conducted to examine the relationship between the delta magnitude of phase advance of core body temperature and the difference in Qmax during the pre-sleep period using Spearman’s correlation. The results indicated a certain trend suggesting an association between the delta core body temperature (dim-bright) and the delta Qmax (dim-bright) (*r* = −0.356, *p* = 0.157); however, this association did not reach statistical significance. Regarding fixed-time urination periods, late-night (04:00) Qmax values were significantly lower than mDay under both bright and dim daylight (*p < 0.05 by two-way repeated measures ANOVA followed by Sidak’s multiple comparison test) (Fig. 3), while the average flow rates or volumes voided per micturition at late night (04:00) were not significantly different versus mDay under each condition (Supplementary Fig. 3).

## Discussion

We observed clear diurnal differences in Qmax in healthy young men under both dim and bright daylight. Morning Qmax values were significantly lower than the bright daylight evening and the daytime, evening, and pre-sleep timepoints in dim daylight. The pre-sleep Qmax values in dim daylight were significantly higher than bright daylight, while late-night Qmax values in the fixed-time urination period were significantly lower than mDay under both the bright and dim daylight conditions.

Diurnal differences in Qmax values were observed in young, healthy men in a light-controlled environment, suggestive of a physiological phenomenon under control of the endogenous central clock system as well as local regulation by bladder-specific timing. The central clock, influenced by light exposure, is located in the supurachiasmatic nucleus and tunes peripheral clocks in organs such as the bladder to coordinate circadian rhythms of intraorgan activities [9]. Qmax is determined by both the contractility of the urinary bladder and resistance of the urethra; greater contractions with lower resistance generate a higher Qmax [25]. Thus, diurnal micturition rhythms are transduced from the circadian clock system [9] to the peripheral bladder clock to prevent urination at night for sound sleep [18]. This shift from voluntary “voiding” mode to “storage” mode at night can be reflected by decreases in bladder contractility.

In addition, we found that bright daylight significantly decreased before sleep Qmax versus dim daylight. This suggests that the pre-sleep bladder may have shifted to “storage” mode under bright daylight conditions, but “voiding” mode might have persisted under dim daylight conditions. In other words, bright daylight could regulate a phase-advancing shift in Qmax diurnal variation, similar to shifts of urinary Na^+^, Cl^−^, uric acid excretion, and rectal temperature rhythms reported by Nakamoto et al. [13]. However, the mechanism of daylight exposure on Qmax pre-sleep is largely unknown. One possible explanation is the advancement of the master clock phase by bright compared with dim light [26]. In such cases, the mode shift of the bladder would have been advanced in line with the advanced master clock signals. Melatonin might precipitate this effect since onset time of melatonin secretion in the evening was reported to be earlier in bright daylight versus dim daylight conditions [27]. In addition to its role in chronobiologic regulation, melatonin also inhibits bladder contraction and is known to increase bladder capacity [28–30]; thus, the early initiation of nocturnal melatonin secretion by bright light exposure during the day may have downregulated Qmax pre-sleep. Given the reported high correlation between core body temperature and melatonin [31], the observed trend in the association between differences in core body temperature and Qmax could align with this hypothesis, despite the limited number of participants.

Psychological influence from the dim daylight condition may have also influenced our results [32, 33]. Since urination is also under voluntary control and this situation was unusual for some participants, it is possible that some participants willingly urinated more strongly than their normal level before going to bed.

In clinical settings, complaints about difficult nighttime urination are common. As a result, one of our objectives was to compare the Qmax at 4 a.m., a time in the middle of the sleep period that is unassociated with urination on demand, with daytime urination. This comparison was a subsidiary study to a previous experiment that focused on measuring urinary components [13]. The data in Supplementary Figure 4 show that, even when participants did not feel the need to urinate based on the fixed interval protocol, there was still a diurnal variation in Qmax, which was lowest in the early morning. Considering that the volume voided per micturition also decreased, it is challenging to determine whether the diurnal variation in Qmax is physiological from this fixed-time urination protocol. This is because Qmax is known to increase as the volume voided per micturition increases and there are significant individual differences in this relationship. Hence, for the statistical analysis of Qmax during scheduled urination, we compared the Qmax during forced urination at 04:00, the actual target time corresponding to sleep, with the value closest to the volume of daytime forced urination.

Diurnal rhythms of urination typically show a decrease in urinary frequency and an increase in volume voided per micturition upon waking or during nighttime [11]. In contrast, our study of healthy young participants revealed diurnal variations in frequency but no such pattern for volume voided per micturition. This could result from the adequate suppression of nocturnal urine production in these young individuals, leading to morning urination before complete filling of the bladder.

The present study has several limitations. First, the origin of diurnal Qmax differences in healthy young men is unreported. While bladder and urethral tension studies in healthy volunteers are possible, invasive catheter insertion is difficult to accomplish [25]. Thus, we could only speculate that melatonin or some psychological effect on bladder/urethral dynamics influenced our observed results. Second, the data obtained in the present study cannot easily be compared with the classical method of constant-routine protocols where participants are in fixed positions and experience sleep deprivation under conditions of constant dim light to determine individual circadian rhythms [34]. Since results from that method are not comparable to real-life situations that may influence circadian patterns, we opted for our novel method, standardizing whatever environmental variables we could; temperature and humidity were constant, while food and water were consumed in fixed quantities and at fixed times. Furthermore, strenuous exercise and napping were prohibited, but no constant positioning or sleep restrictions were imposed [13]. As for the potential masking effect of sleep, a study has shown that Qmax is not related to arousal conditions, as evidenced in newborn experiments [35]. Concerning diet, since elements like caffeine and alcohol are known to influence urination patterns [36], we standardized meal content to control for these dietary effects. Nonetheless, the precise influence of diet on Qmax is still not fully understood. While it remains a challenge to entirely offset the potential masking effects from sleep and diet, our protocol, we believe, effectively captures the variation in Qmax due to inherent diurnal rhythms. Third, the regular urination protocol did not accurately reflect the diurnal variation in Qmax, since it largely depends on bladder capacity and considerable individual differences exist. Therefore, we compared the Qmax at 4 a.m. to daytime values to ensure similar voided volumes. To study the diurnal rhythm of Qmax more precisely, one could monitor the amount of urine accumulating in the bladder continuously and induce urination once a certain volume is reached. However, this method also has limitations: the urination timing might be inconsistent, and there is a continuous need to monitor bladder capacity that could change voluntary voiding choice due to irritation or enhanced bladder focus during measuring. Fourth, concerning average flow rates, there were no diurnal differences observed in the free-urination protocol, nor in the comparison between 4 a.m. and mDay during the regular urination protocol. In light of this, Qmax might be more sensitive than average flow rate, as it more closely reflects the maximal detrusor pressure. We posit that variability in the somewhat lower detrusor pressure possibly remains low between day and night. Further research is required to elucidate this disparity between the diurnal differences of Qmax and average flow rates. Fifth, a standardized comparisons between Qmax and body clock with bright light exposure at night have not yet been reported. Next, only male participants were recruited since complaints of difficult urination at night/early in the morning are uncommon in older women. However, gender differences in circadian rhythms, including renal function, have been reported [37, 38], and we acknowledge that important diurnal Qmax variations may exist between the genders. Finally, the effect of the prostate peripheral clock on this phenomenon remains unknown and a topic for more detailed future studies [39]. In spite of these limitations, we are the first to report Qmax variations in healthy young men that can be used as a baseline to develop further gender-, condition-, and age-based studies.

## Conclusions

Healthy young men had a clear diurnal Qmax difference that decreased during late night and morning. Bright daylight seems to influence this difference pre-sleep by advancing the circadian phase shift, making this process a regulated physiological phenomenon that is instinctive in humans.

### Supplementary Information

**Additional file 1:** Supplementary figures: Supplementary Figure 1. Diurnal differences in frequency and urine production rates during free urination period. Clear diurnal differences in frequency were observed in both bright daylight conditions in (a) and in dim daylight conditions in (b). Clear diurnal differences in urine production rates were also observed both in bright (c) and dim daylight (d) conditions (*p < 0.05, **p < 0.01, ****p < 0.0001 by Dunnett’s multiple comparison test, One-way ANOVA). No significant differences were observed between the Dim and Bright conditions in terms of frequency or urine production rate . by Twoway repeated measures ANOVA. Error bars represent s.e.m. Br indicates bright daylight conditions. Supplementary Figure 2. Diurnal differences in volume voided per micturition and average flow rates during free urination period. No significant diurnal variation of volume voided per micturition was observed in both bright daylight conditions in (a) and in dim daylight conditions in (b). No significant diurnal variation of urine production rates was also observed both in bright (c) and dim daylight (d). No significant differences by One-way ANOVA in a−d. No significant differences were observed between Dim and Bright conditions in volume voided per micturition or average flow rate by Twoway repeated measures ANOVA. Error bars represent s.e.m. Br indicates bright daylight conditions. Supplementary Figure 3. Diurnal differences of average flow rates during fixed-time urination in bright daylight conditions and in dim daylight in (a). mDay: matched Day, indicating closest amount of volume voided per micturition in the daytime compared with 4 a.m. shown in (b). No significant differences were observed between 4 a.m. and mDay in (a) or (b) by Two-way repeated measures ANOVA. Error bars represent s.e.m. Br indicates bright daylight conditions. Supplementary Figure 4. Diurnal variations of Qmax and volume voided per micturition during fixed-time urination under both bright and dim daylight conditions are shown in (a) and (b) respectively. Error bars represent s.e.m. Br indicates bright daylight conditions.

## Data Availability

The datasets generated during and/or analyzed during the current study are not publicly available but are available from the corresponding author on reasonable request.
