# Supplementary material for: Diurnal differences in urine flow in healthy young men in a light-controlled environment: a randomized crossover design
Source: J Physiol Anthropol. 2023 Nov 17;42:27. doi: 10.1186/s40101-023-00346-z (PMC10655426; doi:10.1186/s40101-023-00346-z)
Supplement: Supplementary file 1 — Additional file 1: Supplementary figures: Supplementary Figure 1. Diurnal differences in frequency and urine production rates during free urination period. Clear diurnal differences in frequency were observed in both bright daylight conditions in (a) and in dim daylight conditions in (b). Clear diurnal differences in urine production rates were also observed both in bright (c) and dim daylight (d) conditions (*p < 0.05, **p < 0.01, ****p < 0.0001 by Dunnett’s multiple comparison test, One-way ANOVA). No significant differences were observed between the Dim and Bright conditions in terms of frequency or urine production rate . by Twoway repeated measures ANOVA. Error bars represent s.e.m. Br indicates bright daylight conditions. Supplementary Figure 2. Diurnal differences in volume voided per micturition and average flow rates during free urination period. No significant diurnal variation of volume voided per micturition was observed in both bright daylight conditions in (a) and in dim daylight conditions in (b). No significant diurnal variation of urine production rates was also observed both in bright (c) and dim daylight (d). No significant differences by One-way ANOVA in a−d. No significant differences were observed between Dim and Bright conditions in volume voided per micturition or average flow rate by Twoway repeated measures ANOVA. Error bars represent s.e.m. Br indicates bright daylight conditions. Supplementary Figure 3. Diurnal differences of average flow rates during fixed-time urination in bright daylight conditions and in dim daylight in (a). mDay: matched Day, indicating closest amount of volume voided per micturition in the daytime compared with 4 a.m. shown in (b). No significant differences were observed between 4 a.m. and mDay in (a) or (b) by Two-way repeated measures ANOVA. Error bars represent s.e.m. Br indicates bright daylight conditions. Supplementary Figure 4. Diurnal variations of Qmax and volume voided per micturition during fixed-time ur [file 40101_2023_346_MOESM1_ESM.pdf]

Supplementary Information for

Diurnal differences in urine flow in healthy young men in a light-controlled environment: A randomized cross-over design.

Hiromitsu Negoro[1], Isuzu Nakamoto[2], Sayaka Uiji[3], Yoshiko Matsushima[3], Bryan J. Mathis[4], Dominika Kanikowska[5], Tomoko Wakamura[3]

1Department of Urology, Institute of Medicine, University of Tsukuba, Ibaraki, Japan

2Health Sciences, Graduate School of Medicine, Tohoku University, Miyagi, Japan

3Human Health Sciences, Graduate School of Medicine, Kyoto University, Kyoto, Japan

4International Medical Center, University of Tsukuba Affiliated Hospital, Ibaraki, Japan.

5Department of Pathophysiology, Poznan University of Medical Sciences, Poznan, Poland.

Correspondence should be addressed to Hiromitsu Negoro (E-mail: negoro-jua@umin.ac.jp)

Supplementary Information includes Supplementary Figure 1 to 4

Supplementary Figure 1

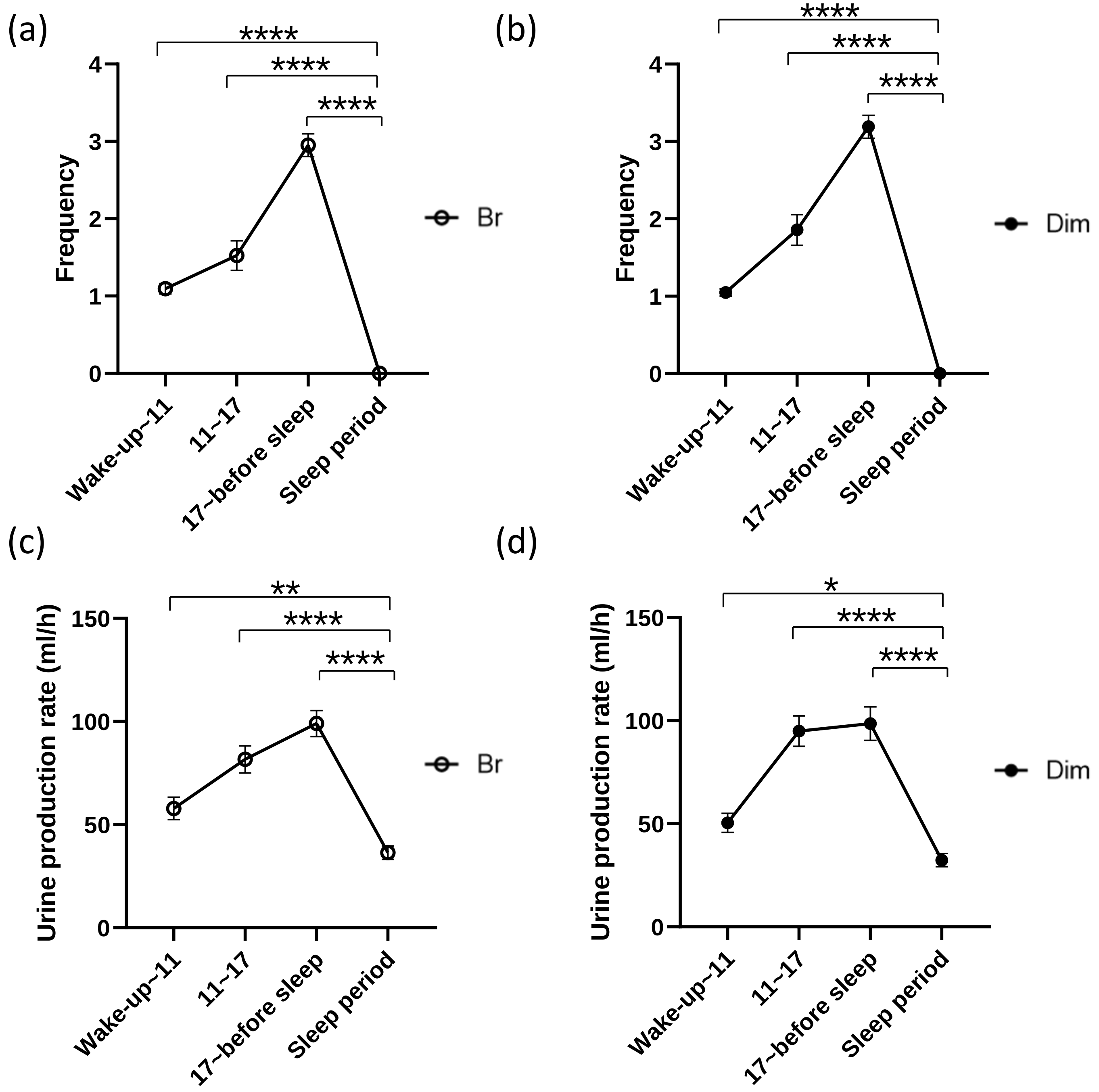

Diurnal differences in frequency and urine production rates during free urination period. Clear diurnal differences in frequency were observed in both bright daylight conditions in (a) and in dim daylight conditions in (b). Clear diurnal differences in urine production rates were also observed both in bright (c) and dim daylight (d) conditions (*p < 0.05, **p < 0.01, ****p < 0.0001 by Dunnett's multiple comparison test, One-way ANOVA). No significant differences were observed between the Dim and Bright conditions in terms of frequency or urine production rate . by Two-way repeated measures ANOVA. Error bars represent s.e.m. Br indicates bright daylight conditions.

Supplementary Figure 2

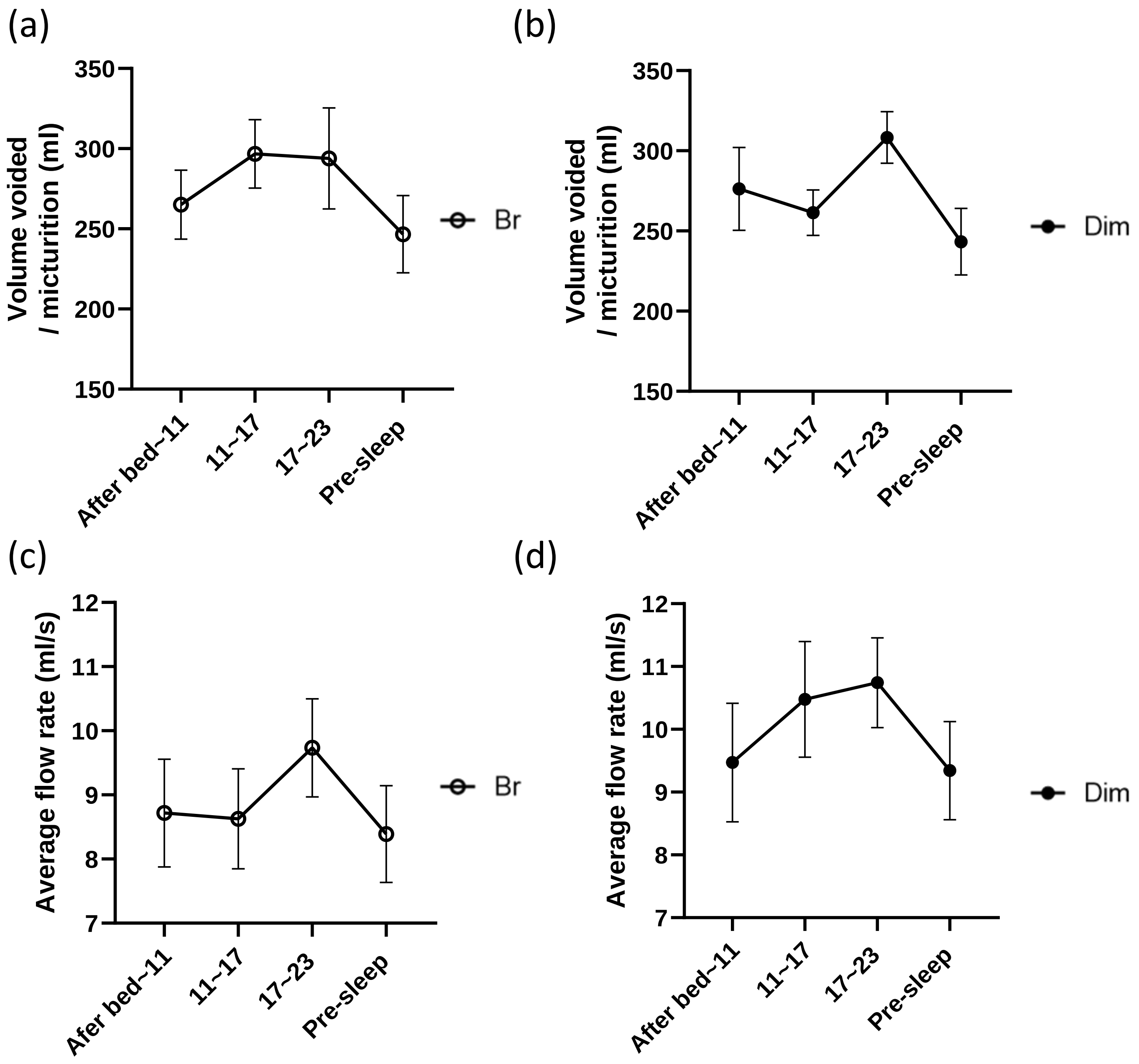

Diurnal differences in volume voided per micturition and average flow rates during free urination period. No significant diurnal variation of volume voided per micturition was observed in both bright daylight conditions in (a) and in dim daylight conditions in (b). No significant diurnal variation of urine production rates was also observed both in bright (c) and dim daylight (d). No significant differences by One-way ANOVA in a–d. No significant differences were observed between Dim and Bright conditions in volume voided per micturition or average flow rate by Two-way repeated measures ANOVA. Error bars represent s.e.m. Br indicates bright daylight conditions.

Supplementary Figure 3

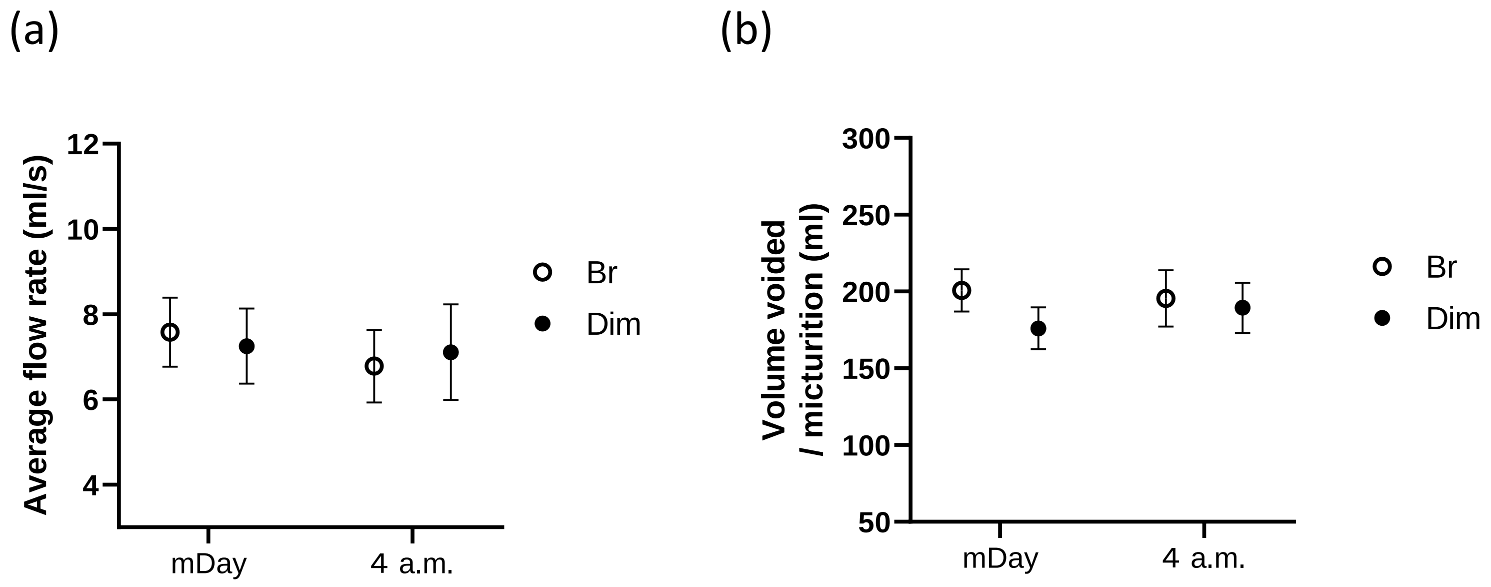

Diurnal differences of average flow rates during fixed-time urination in bright daylight conditions and in dim daylight in (a). mDay: matched Day, indicating closest amount of volume voided per micturition in the daytime compared with 4 a.m. shown in (b). No significant differences were observed between 4 a.m. and mDay in (a) or (b) by Two-way repeated measures ANOVA. Error bars represent s.e.m. Br indicates bright daylight conditions.

Supplementary Figure 4

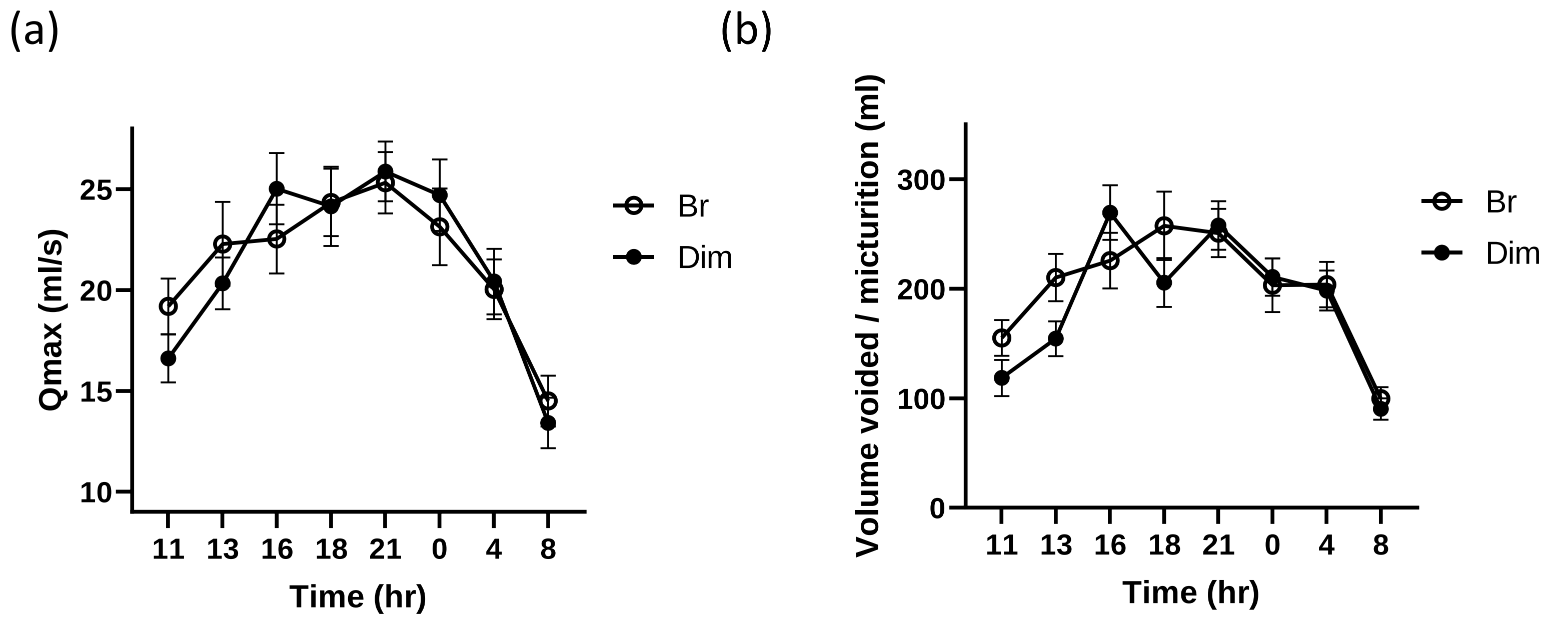

Diurnal variations of Qmax and volume voided per micturition during fixed-time urination under both bright and dim daylight conditions are shown in (a) and (b) respectively. Error bars represent s.e.m. Br indicates bright daylight conditions.
